# Comparison of a minimally invasive posterior approach and the standard posterior approach for total hip arthroplasty A prospective and comparative study

**DOI:** 10.1186/1749-799X-5-46

**Published:** 2010-07-27

**Authors:** Bernd Fink, Alexander Mittelstaedt, Martin S Schulz, Pavol Sebena, Joachim Singer

**Affiliations:** 1 Department of Joint Replacement, General and Rheumatic Orthopaedics, Orthopaedic Clinic Markgröningen gGmbH, Kurt-Lindemann-Weg 10, 71706 Markgröningen, Germany

## Abstract

**Background:**

It is not clear whether total hip arthroplasty performed via a minimally invasive approach leads to less muscle trauma compared to the standard approach.

**Materials and methods:**

To investigate whether a minimally invasive posterior approach for total hip arthroplasty results in lower levels of muscle-derived enzymes and better post-operative clinical results than those obtained with the standard posterolateral approach fifty patients in both groups were compared in a prospective and comparative study. The following parameters were examined: muscle-derived enzymes CPK, CK-MM and myoglobin pre-operatively, 24 and 48 hours post-operatively, CRP and hemoglobin on the third postoperative day, loss of blood, daily pain levels, the rate of recovery (time taken to attain predefined functional parameters), the Oxford Hip Score, the SF-36 score and the WOMAC score pre-operatively and six weeks post-surgery, the position of the implant and the cement coating by post-operative X-ray examination.

**Results and Conclusions:**

The minimally invasive operated patients exhibited a significantly lower loss of blood, significantly less pain at rest and a faster rate of recovery but the clinical chemistry values and the other clinical parameters were comparable.

## Background

A number of different so-called minimally invasive approaches are being used more and more for total hip arthroplasty. In principle they can be divided into two groups: the muscle-sparing approaches and the mini-incision approaches. The former group, where muscles are not cut, includes the two-incision technique, the anterolateral mini-approach and the direct anterior mini approach [1-4]. The mini-incision group approaches involve a shorter incision in the skin and less muscles are detached than in the corresponding standard approach. This group includes the mini-incision lateral approach and the mini-posterior approach [5-8].

In general, the minimally invasive approach is described as having a lower degree of trauma for the soft-tissues and, in particular, for the muscles. This opinion is based on the fact that the loss of blood is lower, rate of recovery is faster, the post-operative level of pain is lower and patients are released sooner from hospital [1-3,8-15]. However, it is unclear whether muscle trauma is really reduced as a result of the smaller sized access incisions and the lack of, or lower amount of, muscle detachment because, normally, the surgical hooks and retractors used during the operation exert a much greater pressure on, and cause extensive contusions in, the muscle tissue. Indeed, measurable muscle damage has been identified in all the currently used minimally invasive approaches tested in cadaver studies [16-18].

The lower level of soft tissue trauma is particularly questionable for the mini-incision techniques. Goldstein et al. [19], Wright et al. [20], Woolson et al. [15] and Ogonda et al. [21] did not observe any objective clinical advantages of the mini-posterior approach when compared to the standard posterolateral approach. It must be said, however, that the minimal invasion in these studies was only at the level of a shorter skin incision. In contrast, Sculco et al. [8,22] and DiGioia et al. [10] observed a smaller loss of blood and a faster post-operative recovery following a mini-posterior approach while Inaba et al. [6] and Dorr et al. [11] reported a lower level of post-operative pain and a more rapid recovery of muscle function using the same technique. The mini-incision technique used in these reports did not involve detachment of the quadratus femoris muscle however.

The objective of the current prospective study was to comparatively analyze not only clinical parameters but also muscle-related clinical chemistry values that could be objectively assessed for the purpose of determining whether the mini-incision posterior approach with its reduced detachment of the external rotator muscles results in a lower degree of muscle trauma than the standard posterolateral approach. Therefore a comparative analysis was performed to answer the question if minimal invasive posterior approach leads to lower muscle enzyme levels, lower postoperative pain, less blood loss and better functional results. Moreover an additional aim was to examine whether the positioning of the implant can be done similarly exact during the two procedures.

## Materials and methods

This report concerns a prospective and comparative study. Fifty patients received a hybrid total hip arthroplasty by means of a mini-posterior (MIS) approach. Fifty patients with the same type of implant implanted via a standard posterolateral (SA) approach were chosen preoperatively so that the two groups were comparable preoperatively according to gender, age, Body Mass Index, ASA score, diagnosis and preoperative Oxford Hip Score (Table 1 and 2). The patients were informed about their kind of surgery.

**Table 1:** Demographic data

Parameter	Standard approach	Mini-posterior approach	p
**Females**	27	25	p = 0.688

**Males**	23	25	p = 0.688

**Age [years]**	71.5 ± 5.6 (61-86)	71.9 ± 6.1 (55-87)	p = 0.737

**BMI [kg/m^2^]**	28.0 ± 3.8 (23-39)	27.0 ± 4.8 (17-40)	p = 0.297

**ASA score [1/2/3]**	3/37/10	4/40/6	p = 0.393

**Table 2:** Laboratorial, clinical and radiographic data

Parameter	Standard approach	**Mini-posterior ap**.	p
**CPK-diff 24 h - preop [U/l]**	569.8 ± 535.1	551.0 ± 295.6	p = 0.829

**CKMM-diff 24 h - preop [U/l]**	553.8 ± 530.0	548.7 ± 290.2	p = 0.952

**Myoglobin-diff 24 h - preop [μg/l]**	205.4 ± 195.0	178.6 ± 143.4	p = 0.336

**CPK-diff 48 h - preop [U/l]**	378.4 ± 218.4	446.3 ± 236.9	p = 0.141

**CKMM-diff 48 h - preop [U/l]**	378.2 ± 218.5	437.4 ± 241.9	p = 0.204

**Myoglobin-diff 48 h - preop [μg/l]**	78.8 ± 88.4	59.9 ± 74.2	p = 0.254

**CRP-diff day 3 - preop [mg/l]**	77.5 ± 38.5	80.1 ± 56.6	p = 0.633

**Operation time [minutes]**	50.9 ± 10.2	51.9 ± 11.4	p = 0.892

**Blood loss intraoperative [ml]**	382.0 ± 179.9	262.7 ± 149.7	p < 0.001

**Blood loss Cell Saver 6 hours postop [ml]**	515.2 ± 348.8	279.0 ± 194.1	p < 0.001

**Blood loss Redon [ml]**	434.0 ± 188.6	352.4 ± 207.2	p = 0.043

**Blood loss total [ml]**	1331.2 ± 538.6	894.2 ± 363.3	p < 0.001

**Retransfusion cellsaver [n of patients]**	13	5	p = 0.037

**Transfusion foreign blood [n of patients]**	3	3	p = 1.0

**Hb-diff day 3 - preop [g/dl]**	3.5 ± 1.57	3.48 ± 1.42	p = 0.953

**Pain at rest [VAS]**	1.11 ± 1.1	0.63 ± 0.67	p = 0.01

**Pain in motion [VAS]**	2.82 ± 1.49	2.57 ± 1.45	p = 0.386

**Mobilisation alone on ward [days]**	3.72 ± 2.03	2.7 ± 1.92	p = 0.049

**Using stairs alone [days]**	6.84 ± 2.35	5.37 ± 1.95	p = 0.011

**Hospital stay [days]**	11.56 ± 3.45	9.96 ± 3.02	p = 0.016

**SF-36 functional score preop**	26.43 ± 11.79	26.37 ± 10.69	p = 0.901

**SF-36 funct. score 6 weeks post**	37.45 ± 15.78	37.53 ± 16.24	p = 0.89

**SF-36 psychological score preop**	43.47 ± 25.87	48.18 ± 27.45	p = 0.523

**SF-36 psych. Score 6 weeks post**	51.39 ± 29.58	52.28 ± 29.62	p = 0.763

**WOMAC preop**	62.7 ± 23.6	60.5 ± 19.8	p = 0.824

**WOMAC 6 weeks postop**	24.1 ± 21.7	22.8 ± 17.2	p = 0.777

**Oxford hip score preop**	41.6 ± 9.2	40.7 ± 6.6	p = 0.665

**Oxford hip score 6 weeks postop**	28.6 ± 10.6	25.5 ± 8.1	p = 0.126

**Cup inclination [degrees]**	42.8 ± 6.6	43.7 ± 5.9	p = 0.583

**Cup anteversion [degrees]**	24.6 ± 4.9	25.1 ± 5.2	p = 0.644

**Stem alignment [degrees in varus]**	0.9 ± 1.2	1.1 ± 1.1	p = 0.682

**Limb length discrepancy [mm]**	0.6 ± 2.7	0.4 ± 1.2	p = 0.581

**Offset [mm]**	2.8 ± 5.2	2.9 ± 4.3	p = 0.931

The exclusion parameters were previous operations on the relevant hip joint, spinal anesthesia (to have the comparable levels of muscle relaxation during the operation) and patients who were not able to comply with the standardized pain medication. The groups consisted of 52 females and 48 males with an average age at the time of the operation of 71.7 ± 5.9 years. The indications requiring endoprosthesis replacement were distributed as follows: 88 cases of osteoarthritis (44 × SA, 44 × MIS), two cases of dysplastic coxarthrosis (1 × SA, 1 × MIS) and 10 cases of femoral head necrosis (5 × SA, 5 × MIS).

**Figure 1:**
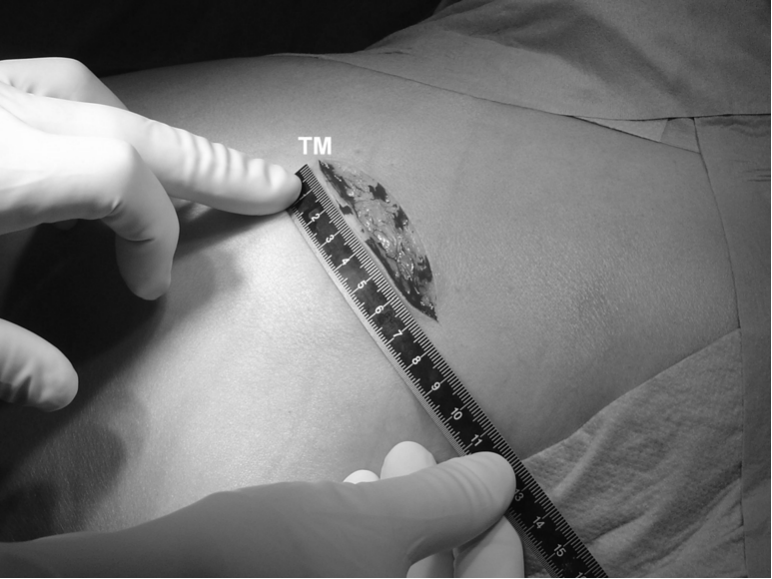
**Skin incision of the minimally invasive posterior approach on a left hip (TM = location of the trochanter major)**.

All patients were implanted with a cementless acetabular press-fit cup [Allofit; Zimmer GmbH, Winterthur, Schweiz] and a cemented stem [Optan; Zimmer GmbH, Winterthur, Schweiz]. The minimally invasive implantation of the hip replacement was carried out by the senior author (B.F.) and involved sparing of the quadratus femoris muscle as described by Inaba et al. [6] among others, although in this case the skin incision was in a different direction (from the posterior edge of the trochanter major in the direction of the fibers of the gluteus maximus; Fig. 1). The implantation of the hip replacement via the standard approach was carried by two of the authors (P.S. 20 hips and J.S. 30 hips) who are both experienced surgeons and perform the operations in the same way, differing only in the length of the skin incision and the extent of the detachment of the external rotator muscles during the operation.

All operations were carried out under intubation anesthesia. A Cell Saver was used in all cases intraoperatively and to remove blood from the operated area via 2 Redon drainage tubes (14 Charrier intra-articular and 12 Charrier subcutaneous) for a period of 6 hours following surgery using a vacuum of 80 cm H_2_O. If more than 600 ml blood was harvested during operation and 6 hours postoperative it was salvaged and re-transfused. Thereafter blood was collected in Redon flasks under vacuum for 42 hours. Blood loss during the operation and during the 6 hours postoperatively were measured whereby the last was calculated using the blood loss in the cell saver in total minus the blood loss intraoperatively. The patients were all given standard pain management treatment that consisted of 1 × Etoricoxib 90 mg (MSD Sharpe & Dohme GmbH, Haar, Germany) daily for 7 days and then this was reduced to Etoricoxib 60 mg once daily, Valoron N 100 1-0-1 (Pfizer Pharma, Karlsruhe, Germany) and Metamizol (Aventis-Pharma Deutschland GmbH, Frankfurt, Germany) 4 × 500 mg daily. Patients who were unable to comply with this pain management treatment because of secondary diseases were excluded from the study.

The clinical chemistry assessment of muscle trauma was carried out pre-operatively, as well as 24 hours and 48 hours after surgery by evaluating myoglobin, using an electrochemiluminescence assay (Elecsys System Modular Analytics E170, Roche Diagnostics, Mannheim, Germany), and both creatine phosphokinase (CPK) and muscle-specific creatine kinase (CK-MM) using an enzyme kinetics method (Elecsys System Modular Analytics E170, Roche Diagnostics, Mannheim, Germany). C-reactive protein (CRP) and hemoglobin (Hb) values were determined pre-operatively and 3 days after surgery. Blood loss, complications and post-operative pain levels (blinded daily measurements using a visual analog scale for pain during rest and during movement) were also recorded. A blind assessment of post-operative recovery was made on a daily basis by recording the mobility of the joint and when the patient was able to walk alone with crutches along the corridor and use stairs without physiotherapist's assistance. Furthermore, the Oxford Hip Score [23], the SF-36 Score [24] and the WOMAC Score [25] were all recorded pre-operatively and then again 6 weeks after surgery. Crutches had to be used for 6 weeks.

Post-operative X-ray images were used to assess the positioning of the implant. Cup inclination was measured from the inter-teardrop line [26]; cup anteversion, with use of the method of Dorr and Wan [27]; and cup fixation, with the method of Udomkiat et al. [28]. Stem alignment was measured on the antero-posterior pelvic radiograph [26], and the quality of the cement of the cemented stems was assessed with the method described by Barrack et al. [29] and Mulroy et al. [30]. Comparison of the limb lengths was based on the distance from the midpoint of the lesser trochanter to the inter-ischial line, and the offset was determined by comparison of the distance from the center of the femoral head to the femoral shaft axis according to Dorr et al. [11]. Clinical examinations were blinded for the examining author (A.M.) with respect to the chosen surgical approach and the radiological assessments blinded for the two assessing authors (A.M. and M.S.). Reliability for the radiographic examinations was high, with an intra-assessor, intra-class correlation coefficient of 0.99 and of 0.98 between assessors, respectively.

The statistical analyses were conducted using the computer program SPSS for Windows (SPSS Inc, Chicago, IL). For comparison between the two groups of surgical approach the Mann-Whitney test was used in the case of quantitative variables. Otherwise, they were compared using the Chi-square test for nominal parameters. The level of significance was fixed at p < 0.05. Institutional review board approval was obtained, and all patients gave their informed consent before participating in this study.

## Results

There was no difference between the increases seen in the post-operative muscle enzyme parameters CPK, CK-MM and myoglobin in either group when compared to the pre-operative values (Table 2). The rise in the CRP values was also comparable in both groups (Table 2).

In contrast, there was a significantly lower loss of blood in the MIS group, not only in the intra-operative phase but also in the period up to removal of the Redon drainage tubes (Table 2). In parallel, the standard approach group contained 8 patients who exhibited wound secretion beyond the 7^th ^post-operative day whereas the MIS-group only contained 1 such patient (p = 0.014). This leads to a longer mean hospital stay for the standard group compared to the MIS-group (Table 2). Blood retransfusions of the cellsaver were given more often in the SA-group than in the MIS-group, foreign blood transfusion to both groups at the same rate and there was no difference in Hb-values recorded on the third post-operative day and the pre-operative measurements (Table 2).

**Figure 2:**
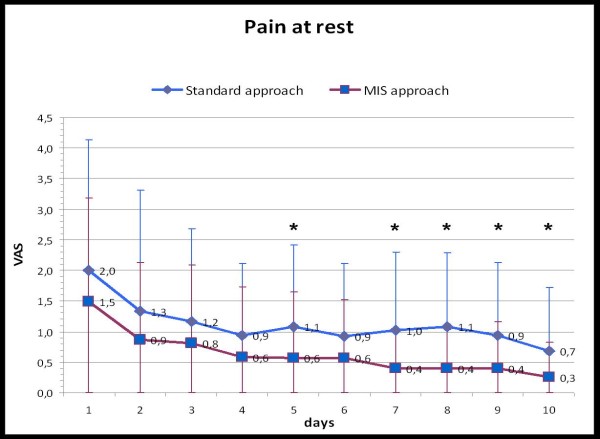
Development of the pain at rest after the operation for both approaches (VAS = visual analog scale, * = significant differences).

**Figure 3:**
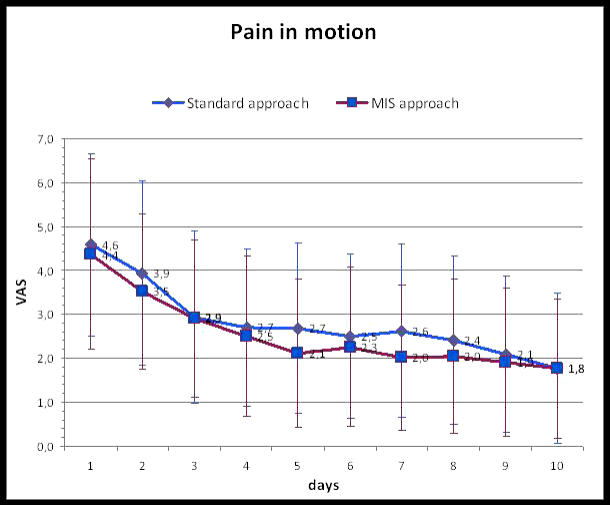
**Development of the pain in motion after the operation for both approaches (VAS = visual analog scale)**.

From a clinical point of view, the patients in the MIS-group reported significantly less pain at rest but not during movement (Table 2, Fig. 2, 3). This difference in resting pain levels became apparent from the fifth post-operative day (Fig. 2).

As far as rate of recovery was concerned, the patients in the MIS-group were able to walk along the corridor and climb stairs unassisted at significantly earlier times than the SA-group (Table 2). There were no differences in the Oxford Hip Score, the SF-36 Score or the WOMAC Score when assessed 6 weeks after surgery.

The evaluation of the X-ray images did not reveal any differences in any of the parameters used for assessing the two groups (Table 2); in particular, the MIS-group did not exhibit a more frequent malpositioning of the implant. The cement mantle was complete in all cases as classified according to Barrack et al. [29] and Mulroy et al. [30]. Apart from one dislocation reported for each group, both of which then underwent closed repositioning, there were no further complications such as fracture, nerve lesions, infections or deep vein thrombosis.

## Discussion

The value of minimally invasive surgery for hip arthroplasty is still unclear. The gait analyses by Dorr et al. [11]. and the investigation of post-operative mobilization by DiGioia et al. [10] suggest that there is a reduction in the degree of muscle traumatization but this could not be confirmed by the analysis of muscle-associated enzymes described in this report. This supports the findings of Suzuki et al. [31] who also failed to observe any significant differences in levels of CPK following mini-posterior and standard posterior approach surgery. Although it is generally accepted that the level of the muscle-related enzymes and proteins examined, i.e. CPK, CK-MM and myoglobin, are markers for the degree of muscle trauma after injury, it is not absolutely clear whether these parameters are meaningful for the situation following surgical trauma of the muscles [32-34]. This is suggested by the high level of variability of the muscle enzyme values with very different individual values observed within our study and in the study of Cohen et al. [35] who did not find differences in muscle enzymes comparing the mini posterior, mini modified Watson Jones approach and a mini double incision approach. If one accepts that the muscle enzyme values are meaningful parameters, then this could mean on one hand that the degree of trauma associated with minimally invasive and standard posterior approaches to the surgery is the same in both cases. This could be explained by the fact that although the minimally invasive technique has a lower degree of muscle trauma because fewer sharp instruments are used and there is less detachment and incision of the muscle, this is balanced out by the use of hooks and retractors to expose the operation site, which in itself causes blunt trauma. This explanation is supported by cadaver studies which have shown that measurable muscle damage occurs during the mini-posterior approach as well as during all the currently practiced minimally invasive techniques [16-18]. On the other hand similar muscle enzyme levels in both groups could be explained by the fact that myocyte stress is similar in both groups but the additional detachment of muscles from bone in the standard approach lead to additional damage of the muscle without additional elevation of enzyme levels but functional worse results in the early postoperative period.

In addition, our data does not support the conclusion drawn by Suzuki et al. [31] from their clinical chemistry studies in which they identified significantly lower levels of CRP in the minimally invasive group than in the standard posterolateral group and concluded that there was a reduced post-operative inflammatory reaction in the minimally invasive approach group.

In contrast the observation of smaller amounts of blood loss reported by Sculco et al. [8] could be confirmed by the results of this study. This could be explained by the fact that the minimally invasive approach not only results in a smaller wound size but also involves detachment of only the upper part of the external rotator muscles, so sparing the rami profundus of the circumflexa femoris medialis artery. The comparable Hb-levels in both groups can be explained by retransfusion of cell-saver blood which was done significantly more often in the SA-group. Therefore in our study the Hb-level is not a good parameter for blood loss due to the surgery. The smaller wound in the MIS group may also be responsible for the lower levels of post-operative pain that we and others observed in the MIS group [6,11].

The significantly earlier ability to walk alone in the corridor and to climb stairs unassisted illustrates the benefit of the minimally invasive approach with respect to the post-operative recovery period. This advantage was also reported by other authors [6,11]. However, a bias can not be excluded because the patient in our and in other studies were informed about the kind of their surgery which may result in higher motivation of patients of the MIS group. Six weeks after surgery the clinical scores in our study and in the report of Dorr et al. [11] showed no longer any differences so that there does not appear to be a benefit for longer term of minimally invasive surgery. This was also confirmed by gait analyses which showed that there was no difference between the mini-posterior approach and the standard posterior approach 6 weeks after implantation of hip endoprostheses [11,36].

A weakness of this study is clearly the lack of any randomization of the patients which may bias the results. However, the primary objective of this study was to assemble a non-selected group of patients with as few exclusion criteria as possible and to avoid the exclusion of a number of patients because they wished to undergo minimally invasive surgery. This corresponds to procedures described in other studies that compared various minimally invasive approaches and the standard approach to total hip replacement [6,15,20,37,38]. Furthermore, the fact that two different surgeons performed the implantations via the standard approach may bias the results. However, all three surgeons were well experienced and the operative procedure was exactly the same except the shorter incision and the preserving of the lower external rotators in the minimal invasive group. In the standard approach both experienced surgeons did exactly every step identical and there was no difference in the results between them. Moreover, the patients were not entered into a post-operative recovery program especially designed for minimally invasive surgery patients as they were in the study of Dorr et al. [11] Instead, it was decided to examine whether an unchanged rehabilitation program would result in the minimally invasive surgery group attaining defined rehabilitation objectives at an earlier time and so avoid the mixing of the effect of a different rehabilitation program with the effect of the surgical approach. Moreover, the fact that patients with the minimal invasive approach know that they get this kind of approach may bias the results, but this is the problem in all studies analysing minimal-invasive approaches.

## Conclusions

Thus it can be concluded that the minimally invasive posterior approach has a demonstrable advantage over the standard posterior approach during the implantation of hip endoprostheses in that there is lower loss of blood, less post-operative pain and a more immediate post-operative recovery. It was not possible to demonstrate a lower degree of muscle trauma on the basis of muscle-associated enzymes, however, so it is questionable whether muscle enzymes do reflect the muscle trauma or whether the positive effect of the minimally invasive approach during the early post-operative phase is a function of the degree of muscle trauma at all. This and previous studies have shown that the minimally invasive technique results in a reproducibly good positioning of the implant and optimal cementing technique and is not associated with higher complication rates than the standard approach. The minimally invasive surgical approach thus represents a viable option for the implantation of hip endoprostheses.

## Competing interests

The authors declare that they have no competing interests.

## Authors' contributions

BF conceived of the study, participated in its design and coordination and drafted the manuscript

AM participated in the study and analyses of the study

MSS participated in the design of the study and performed the statistical analysis

PS participated in the study and analyses of the study

JS participated in the study and analyses of the study

All authors read and approved the final manuscript.
